# Neuroprotective and intraocular pressure lowering effects of dual-functional memantine nitrate MN-08 on the experimental models of glaucoma

**DOI:** 10.1038/s41598-025-06832-x

**Published:** 2025-07-03

**Authors:** Huihui Hu, Liangmiao Wu, Xiaodie Hu, Minghua Wang, Yewei Sun, Gaoxiao Zhang, Yuqiang Wang, Peng Yi, Zaijun Zhang

**Affiliations:** 1 Department of Intensive Care Unit, The First Affiliated Hospital of Jinan University and Institute of New Drug Research, Jinan University College of Pharmacy, Guangzhou, 511436 China; 2 State Key Laboratory of Bioactive Molecules and Druggability Assessment, and Guangzhou Key Laboratory of Innovative Chemical Drug Research in Cardio-Cerebrovascular Diseases, and Institute of New Drug Research, Jinan University, Guangzhou, 511436 China; 3 Guangdong-Hong Kong-Macau Joint Laboratory for Pharmacodynamic Constituents of TCM and New Drugs Research, and Guangdong Province Key Laboratory of Pharmacodynamic Constituents of TCM and New Drugs Research, Jinan University College of Pharmacy, Guangzhou, 511436 China; 4 International Cooperative Laboratory of Traditional Chinese Medicine Modernization and Innovative Drug Development of Chinese Ministry of Education (MOE), Jinan University College of Pharmacy, Guangzhou, 511436 China; 5 Department of Neurology, Daqing People’s Hospital, Daqing, 163319 China

**Keywords:** Glaucoma, Memantine nitrate, Intraocular pressure, Nitric oxide, Ocular hypertension, Neurology

## Abstract

Glaucoma stands out as the primary cause of permanent blindness worldwide, marked by elevated intraocular pressure (IOP) and the deterioration of retinal ganglion cells (RGCs) within the optic nerve. This study aimed to investigate the therapeutic efficacy of MN-08, a novel memantine nitrate derivative, in experimental models of glaucoma. In the rat model of retinal ischemia–reperfusion injury, MN-08 prevented the reduction in retinal ganglion cell complex (GCC) thickness and RGC loss. Meanwhile, MN-08 reduced the protein levels of cleaved-caspase-3, caspase-3, and Bax, while increasing the expression of Bcl-2 compared to the model group, indicating that MN-08 exerts an inhibitory effect on the apoptosis of RGCs. Furthermore, MN-08 lowered IOP in the transient ocular hypertension and glaucoma rabbit models. MN-08 significantly increased cGMP levels in human trabecular meshwork cells (HTMCs). Elevation of cGMP induced by MN-08 was eliminated by ODQ. Moreover, MN-08 attenuated the protein levels of MLCK and p-MLC-2 in HTMCs induced by endothelin-1. Additionally, MN-08 exhibited favorable distribution profiles and pharmacokinetic parameters in normal rabbit ocular tissues following topical instillation. These findings indicate that MN-08 could antagonize NMDA receptors to protect RGCs and release NO to relax TM for the treatment of glaucoma.

**Supplementary Information:**

The online version contains supplementary material available at 10.1038/s41598-025-06832-x.

## Introduction

Glaucoma is a chronic, degenerative optic neuropathy that originates with high intraocular pressure-induced death of retinal ganglion cells (RGCs) and subsequent damage at the optic nerve head, which ultimately leads to visual field defects. It is one of the most prevalent causes of irreversible blindness globally, affecting approximately 80 million individuals^1,2^. Alarmingly, about 50% of glaucoma patients in developed countries remain undiagnosed. Unfortunately, current therapeutic options for glaucoma are limited. There are four classes of agents available for its treatment: prostaglandins, beta-blockers, carbonic anhydrase inhibitors, and alpha-2 agonists^3^. These agents primarily focus on lowering intraocular pressure and have no protective benefits against RGC loss, as each drug targets only a single mechanism. Therefore, there is an urgent necessity for more effective and multi-functional therapeutic agents.

High intraocular pressure (IOP) is closely linked to the initiation and advancement of glaucoma. There is a 10% increase in the risk of the disease developing and occurring for every millimeter of mercury increase in IOP, according to several randomized controlled clinical trials^4^. Currently, effectively controlling or reducing IOP is one of the most critical strategies to stop or slow the progression of glaucoma. Normal intraocular pressure in healthy human eyes ranges from 10 to 20 mmHg and is maintained by the balance between the rate of aqueous humor (AH) production and its drainage through the outflow pathway. Under healthy physiological conditions, the outflow of AH is primarily accomplished through the conventional pathway, which includes Schlemm’s canal and trabecular meshwork (TM), while the uveoscleral or nonconventional pathway contributes to a lesser extent. Current therapies to lower IOP include prostaglandin analogs, which are thought to lower IOP by influencing the uveoscleral pathway, as well as carbonic anhydrase inhibitors, α-adrenergic receptor agonists, and β-adrenergic receptor blockers, all of which target aqueous humor formation (AHF)^5^. Converging evidence suggests that the nitric oxide (NO)-cGMP signaling pathway may serve as an emerging target for regulating aqueous humor dynamics and IOP in the context of treating ocular hypertension (OHT) and glaucoma^6,7^. NO-induced IOP reduction may mediate pressure by regulating the conventional pathway, leading to a significant enhancement in the aqueous outflow. In a nonhuman primate’s study, NO donors and the cGMP analog (8-Br-cGMP) resulted in a 60–90% increase in outflow when administered via topical or intravitreal injection^8,9^. Furthermore, the effect of NO-donating compounds on IOP has been investigated across several species, including monkeys, dogs, rabbits, and mice. In a normotensive rabbit model, the topical administration of NO-donating compounds such as nitroglycerin, sodium nitroprusside (SNP), sodium nitrite, and isosorbide dinitrate (ISDN) rapidly reduced IOP, reaching peak values within 1–2 h post-treatment^10,11^. Latanoprostene bunod (LBN), a prostaglandin F2α receptor agonist that donates NO, has been shown to achieve a great reduction in IOP compared to latanoprost (an FDA-approved first-line IOP-lowering agent) in both preclinical models and glaucoma patients^12–14^. Notably, in 2017, the FDA approved LBN to reduce IOP in patients with ocular hypertension or open-angle glaucoma. Thus, topical administration of NO donors presents a promising approach for glaucoma patients by effectively lowering IOP through the targeted modulation of the diseased conventional outflow pathway.

Although elevated IOP is acknowledged as the primary risk factor for glaucoma progression, traditional agents for reducing IOP have been incapable of halting or reversing progressive RGCs loss and vision defects in some glaucoma patients^15^. A developing field of glaucoma research is therefore neuroprotection, which has been suggested as a supplementary therapeutic avenue, both independent of and complementary to IOP-decreasing treatments. This approach focuses on directly targeting the neurons of the visual centers and RGCs^16–18^. Glutamate‑induced excitotoxicity, oxidative stress, and elevated IOP are all closely related to the RGCs death in glaucoma animal models and patients^19–23^. Increasing evidence shows that elevated glutamate concentrations induced by *N*-methyl-D-aspartate (NMDA) receptor hyperactivation, sufficient to damage the RGCs, have been detected in the vitreous humor of individuals with glaucoma as well as in glaucoma animal models^24,25^. Subcutaneous or intravitreal injections of glutamate selectively disrupted the inner layer of the retina, which contains the RGCs. Based on these observations, reduction of glutamate activity through inhibiting the NMDA receptors has been considered a crucial strategy for RGCs protection. Memantine, a non-competitive NMDA receptor antagonist that functions as an open-channel blocker with moderate binding affinity, was tested for efficacy as a neuroprotective agent for glaucoma in a phase 3 clinical trial^26^. Although memantine had no significant effect in protecting visual function, its beneficial effect on RGCs has been confirmed in both preclinical and clinical studies^26–28^.

The failure of memantine in phase 3 clinical trials may be mainly due to its single functional target, whereas glaucoma is a multifactorial disease. Therefore, the capacity of a single medication to achieve both IOP reduction and neuroprotection may be the key to breakthrough therapy for glaucoma, a condition that requires a comprehensive approach. Based on the above, we designed and synthesized memantine nitrate by inserting a nitrates group into the memantine backbone, and thought that these memantine nitrates could bind to NMDA receptors through their memantine scaffolds. Targeted approaches deliver NO to pathologically open NMDA coupled channels^29–31^. Recently, we have demonstrated that the innovative compound MN-08 simultaneously inhibits NMDA receptors and releases NO, with both neuroprotective and vasodilatory effects^32–34^. Furthermore, dual‐functional MN‐08 exhibits significant therapeutic effects across a variety of preclinical animal models of diseases, including Alzheimer’s disease (AD), subarachnoid hemorrhage (SAH), pulmonary arterial hypertension (PAH), and acute lung injury^32,33,35,36^. However, it is not clear whether MN-08 could play the dual role of neuroprotection and IOP-lowering by antagonizing NMDA receptors to protect RGCs and releasing NO to relax TM for the treatment of glaucoma. In the current study, we examined the efficacy of MN-08 in experimental glaucoma models and further investigated its mechanisms of action.

## Materials and methods

### Animals

Male Sprague Dawley rats (240–250 g) were sourced from the Guangdong Medical Laboratory Animal Center (Guangzhou, China), and New Zealand rabbits (2.0–2.5 kg) were procured from the Guangzhou Xinhua Experimental Animal Breeding Center (Guangzhou, China). Animals were kept in standard laboratory conditions with a room temperature maintained between 20 and 25 °C and a 12 h light/dark cycle. All animal procedures were performed in accordance with the National Research Council’s Guide for the Care and Use of Laboratory Animals Science, which complied with the ARRIVE guidelines and U.S. National Institutes of Health (NIH) Guide for the Care and Use of Laboratory Animals. All experimental procedures involving the animals received approval from the Institutional Animal Care and Use Committee (IACUC) of the Guangzhou University of Chinese Medicine (Guangzhou, China, No. 202302005).

### Retinal ischemia–reperfusion injury model in rats

Male Sprague Dawley rats were anesthetized with pentobarbital sodium (30 mg/kg, i.p.). Local anesthesia was administered to the corneas using tetracaine hydrochloride, followed by pupil dilation with tropicamide. A 30-gauge infusion needle was inserted into the anterior chamber of the eye and connected to a normal saline reservoir until the iris turned white. Retinal ischemia was maintained for 1 h at IOP of 110 mmHg. Upon careful withdrawal of the needle, IOP returned to normal, thereby inducing retinal ischemia–reperfusion injury. MN-08 (12 mg/kg) and Memantine (10 mg/kg) were administered via tail vein injection at 3 and 6 h post-surgery and continued twice daily for the subsequent two days. The rats were sacrificed on the fourth day.

### Transient ocular hypertension model in rabbits

The methods were conducted in a previous study^37^. New Zealand rabbits were anesthetized using an auricular vein injection of 60 mg/kg pentobarbital sodium. The corneas were under local anesthesia with tetracaine hydrochloride, followed by pupil dilation with tropicamide. Subsequently, rabbits received a 100 μL injection of hypertonic saline solution (5% NaCl) into the vitreous body of both eyes to induce a transient IOP elevation model. MN-08 (concentrations of 0.5%, 0.2%, 0.1%, and 0.02%) or latanoprost (0.005%, latanoprost ophthalmic solution, Pfizer) were then respectively instilled in a total volume of 50 μL into the conjunctival pocket of each eye after hypertonic saline solution injection. IOP was measured using a TONOLAB rebound tonometer (iCare) at baseline (time 0) and subsequently at 10, 30, 60, 90, 120, 150, and 180 min post-injection (with 5 consecutive measurements being averaged to obtain a single value). At the last IOP measurement, the rabbits were euthanized by intravenous injection of pentobarbital sodium (100 mg/kg).

### Carbomer-induced experimental glaucoma model in rabbits

The methods were performed as previously described^38^. New Zealand rabbits were anesthetized using an auricular vein injection of 60 mg/kg pentobarbital sodium. Local anesthesia of the corneas was achieved using tetracaine hydrochloride, followed by pupil dilation with tropicamide. An experimental glaucoma model was established by bilaterally injecting 0.25% carbomer (0.1 mL) into the anterior eye chamber of the rabbits. On the fifth day post-carbomer injection, stable high IOP was induced in the rabbits. MN-08 (0.5%, 0.2%, 0.1%, and 0.02%) or latanoprost (0.005%) was administered with a single topical instillation. IOP was measured continuously at 0, 30, 60, 120, 240, and 360 min following instillation with MN-08 or latanoprost (with 5 consecutive measurements being averaged to obtain a single value). At the last IOP measurement, the rabbits were euthanized under sodium pentobarbital anesthesia (100 mg/kg, i.v).

### Cell culture

Primary human trabecular meshwork cells (HTMCs) were isolated from the juxtacanalicular and corneoscleral regions of the human eye from ScienCell Research Laboratories (ScienCell, Carlsbad, CA). HTMCs were maintained in a complete medium consisting of DMEM enriched with 10% fetal bovine serum (FBS) and antibiotics (100 U/ml penicillin and 100 μg/ml streptomycin). Cells were cultured in a humidified incubator containing 5% CO_2_ at 37 °C. Experiments are conducted using HTMCs between the 5th and 8th passages to avoid cellular senescence. All reagents were obtained from Gibco (San Diego, CA, USA).

### cGMP ELISA assay

HTMCs were seeded at a density of 2 × 10^5^ cells per well in 6-well plates. When reaching 80% confluency, the cells were subsequently incubated overnight in low-serum medium (DMEM supplemented with 0.5% FBS). Cells were pretreated with ODQ (a selective soluble guanylyl cyclase (sGC) inhibitor, 1 μM) for 30 min and then exposed to MN-08 (5, 10, and 20 μM), Memantine (10 μM), or latanoprost (0.1 μM) for 30 min. After incubating, cells were harvested and lysed, and the levels of cGMP were detected by an ELISA kit (Abcam Corporation, Cambridge, UK). The procedure was conducted following the instructions provided by the manufacturer.

### HE staining

Rats were euthanized by deep anesthetization with pentobarbital sodium (100 mg/kg, i.p.), and their eyes were enucleated for histology analysis. Eyes were preserved in 4% PFA overnight and subsequently embedded in paraffin. Following sectioning, retinal slices (5 µm) were stained using hematoxylin and eosin (H&E). The morphology and structure of the retina were observed at 400 × magnification by Light microscopy (Olympus, Tokyo, Japan). Ganglion cell complex (GCC) thickness and the RGCs numbers (retinal ganglion cell layer) were measured and analyzed with ImageJ software.

### Immunofluorescence staining

Eyes of sacrificed rats were rapidly removed; the cornea was cut along the corneoscleral rim after rinsing with PBS under the microscope. The lens and vitreous body were separated with micro forceps, the anterior segment of the eye was cut, and the retinal cup was immersed in 4% paraformaldehyde for 1 h. After routine gradient dehydration, four incisions were carefully made in the retina, which was spread flat on the glass slide. The retinal flat mounts were blocked in 5% BSA (containing 2% Triton X-100) at room temperature for 1 h, followed by incubating with primary antibodies against Brn-3a (Abcam, ab245230, 1:200) overnight at 4 °C. Retinas were then rinsed with PBS three times and incubated with fluorescently labeled secondary antibodies (Thermo, A32733, 1:1000) in the dark for 1 h. RGCs were observed using a fluorescence microscope (Olympus, Tokyo, Japan). The number of Brn-3a positive cells was counted manually in five randomly selected fields of view for each region of the whole-mount retina (including the center, mid-periphery, and periphery).

### Western blotting

Eye tissues or HTMCs were lysed using cold RIPA buffer (Beyotime, P0013) containing protease and phosphatase inhibitor cocktail I (MCE, HY-K0010, HY-K0021). BCA assay (Fude Biological Technology Co., Ltd., FD2001) was employed to quantify protein concentrations. Proteins underwent separation using 10% SDS-PAGE before being transferred to PVDF membranes (Merck, ISEQ00010). After incubation for 1.5 h with a blocking solution composed of 5% skim milk, membranes were subsequently incubated overnight at 4 °C with primary antibodies against Bcl-2 (CST, 2764s), Bax (CST, 2772s), Caspase-3 (CST, 9662s), Cleaved caspase-3 (CST, 9664s), MLCK (Abcam, ab232949), p-MLC-2 (CST, 3671s) and β-actin (CST, 4967s). The PVDF membranes were then incubated with either an anti-rabbit IgG HRP-linked antibody (CST, 7074s) or an anti-mouse IgG HRP-linked antibody (CST, 7076s) for 1.5 h at room temperature. The protein band was visualized through chemiluminescence, and protein levels were quantified with Carestream MI SE analysis software.

### Tissue distribution and pharmacokinetic parameters of MN-08 in rabbit ocular tissues

Twenty-four adult New Zealand rabbits, comprising an equal number of males and females, with a weight of 1.8–2.3 kg, were utilized in this experiment. The rabbits were randomly assigned to four groups (n = 6 per group). MN-08 was dripped into the left and right eyes at a concentration of 0.25 mg/50 μL. The rabbits were anesthetized with pentobarbital sodium (60 mg/kg) at 0.1, 1, 4, and 8 h after administration. Blood was collected from the central ear artery into EDTA-K_2_ tubes and centrifuged at 3000 rpm for 10 min to separate the plasma. Both eyeballs were stripped (three rabbits of each sex at each sampling point), followed by the aspiration of aqueous humor, as well as the conjunctiva, cornea, iris-ciliary body (ICB), lower eyelid, optic nerve, retina, and sclera. Aqueous humor was promptly acidified by adding an equal mass of 0.13 M HCl. The separated tissues were snap-frozen in liquid nitrogen and stored in a -80 °C refrigerator until analysis. The concentration of MN-08 was quantified in the ocular tissues and plasma of rabbits using LC–MS/MS analytics.

### Statistical analysis

All data were presented as means ± SEM, and all experiments were repeated at least three times. One-way ANOVA followed by Dunnett’s test or two-way ANOVA followed by Tukey’s test was performed using GraphPad Prism 8 software, with a *p*-value < 0.05 regarded to be statistically significant.

## Results

### MN-08 prevents RGCs loss in retinal ischemia–reperfusion injury rats

**Fig. 1:**
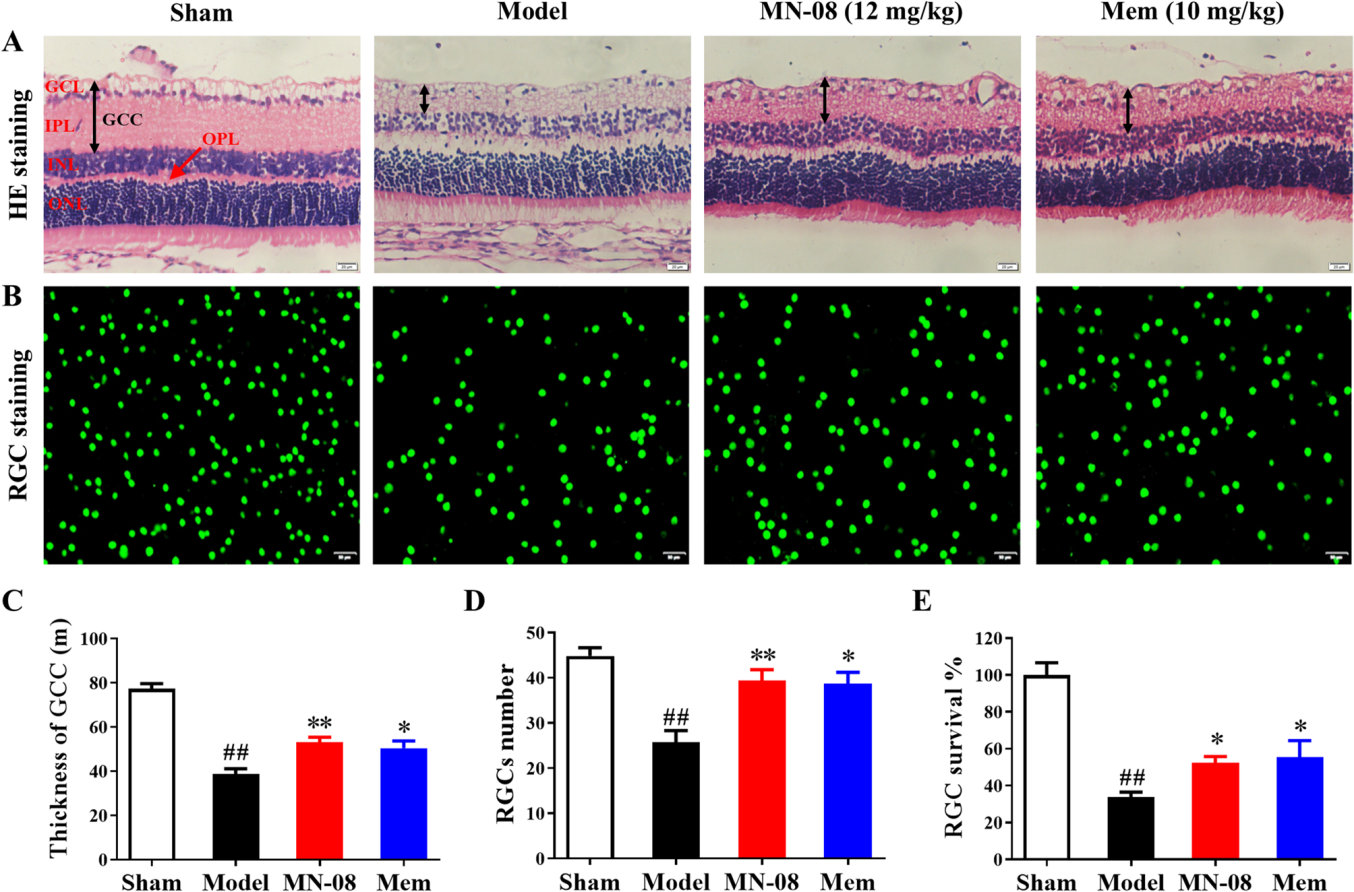
MN-08 prevents RGCs loss in retinal ischemia–reperfusion injury rats. (**A**) Representative histological images of retinal sections stained with H&E. The double black arrow indicates the thickness of GCC. The red arrow indicates the outer plexiform layer. Scale bar: 50 μm. (**B**) Retinal flat mounts were immunolabeled with Brn-3a antibodies and examined using a fluorescence microscope. Scale bar: 50 μm. GCC thickness (**C**), RGCs numbers in GCL (D), and RGCs survival rate (**E**) were analyzed and measured with ImageJ software in each group. Data were presented as the means ± SEM (n = 5). ^*##*^*P* < 0.01 versus Sham group; *P < 0.05 versus Model group; **P < 0.01 versus Model group.

We first established a retinal ischemia–reperfusion injury rat model using saline anterior chamber perfusion. HE staining was employed to assess the RGCs loss and GCC thickness after retinal ischemia–reperfusion injury in rats. The retina is a multilaminar structure composed of three layers of cells: the outer nuclear layer (ONL), the inner nuclear layer (INL), and the ganglion cell layer (GCL). As shown in Fig. 1A, the retinal layers in the sham group were well arranged, while the ONL and INL cells in the model group were loosely arranged, the thickness of the inner plexiform layer (IPL) was significantly thinner, and the number of ganglion cells was significantly reduced. The degree of retinal damage was reduced after MN-08 and Memantine treatment compared with that in the Model group. The arrangement of cells in the outer nuclear layer and inner nuclear layer was slightly disordered. Additionally, immunolabelling was performed with an antibody to Brn-3a, a marker that has been used to identify RGCs in the retina. As illustrated in Fig. 1B, the survival rate of RGCs was markedly decreased in the model group. MN-08 and Memantine treatment significantly increased the survival of RGCs. Taken together, these results indicated that MN-08 treatment reduces retinal damage and RGCs loss in the retinal ischemia–reperfusion injury rats (Fig. 1C–E).

### MN-08 reverses the altered expression of apoptosis-related proteins in retinal ischemia–reperfusion injury rats

**Fig. 2:**
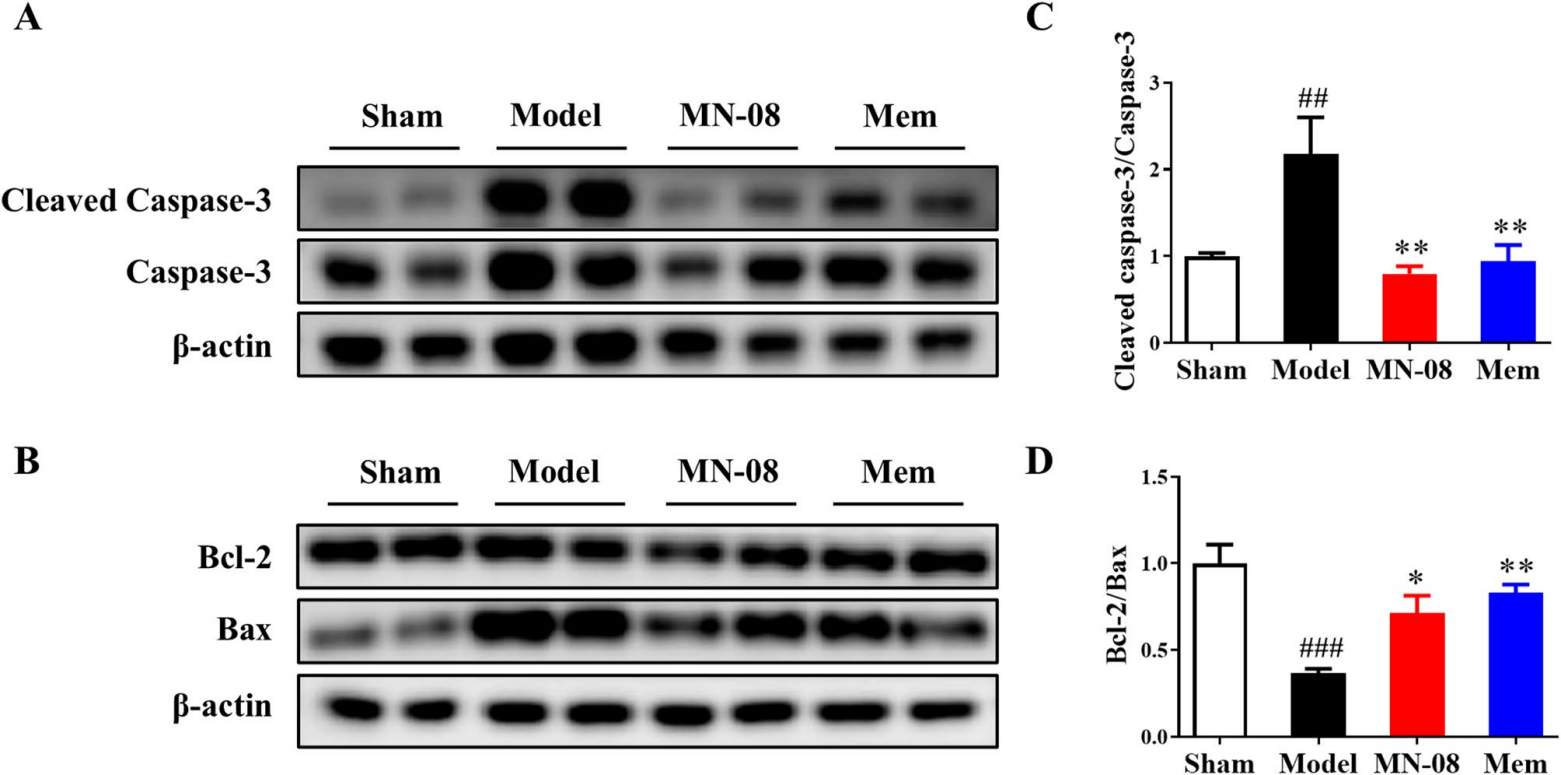
MN-08 reverses the altered expression of apoptosis-related proteins in retinal ischemia–reperfusion injury rats. (**A**–**B**) Representative images of the protein expression of cleaved caspase-3, caspase-3, Bcl-2, Bax, and β-actin. (**C**–**D**) Quantitative analysis of cleaved caspase-3/caspase-3 and Bcl-2/Bax expression. Data were presented as the means ± SEM (n = 4). ^*##*^*P* < 0.01 versus Sham group; ^*###*^*P* < 0.001 versus Sham group; ^*^P < 0.05 versus Model group; **P < 0.01 versus Model group.

We evaluated the protein expression levels of cleaved-caspase-3, caspase-3, Bcl-2, and Bax in retinas by Western blot. As shown in Fig. 2A–B, the retinal ischemia–reperfusion injury rats exhibited elevated levels of the apoptotic proteins cleaved-caspase-3, caspase-3, and Bax, alongside reduced expression of the anti-apoptotic protein Bcl-2. MN-08 treatment decreased the expression of cleaved-caspase-3, caspase-3, and Bax, while simultaneously increasing the expression of Bcl-2 compared to the model group (Fig. 2C–D). These results suggest that MN-08 exerts an inhibitory effect on retinal neuron apoptosis.

### MN-08 reduces IOP in the transient ocular hypertension rabbit model

**Fig. 3:**
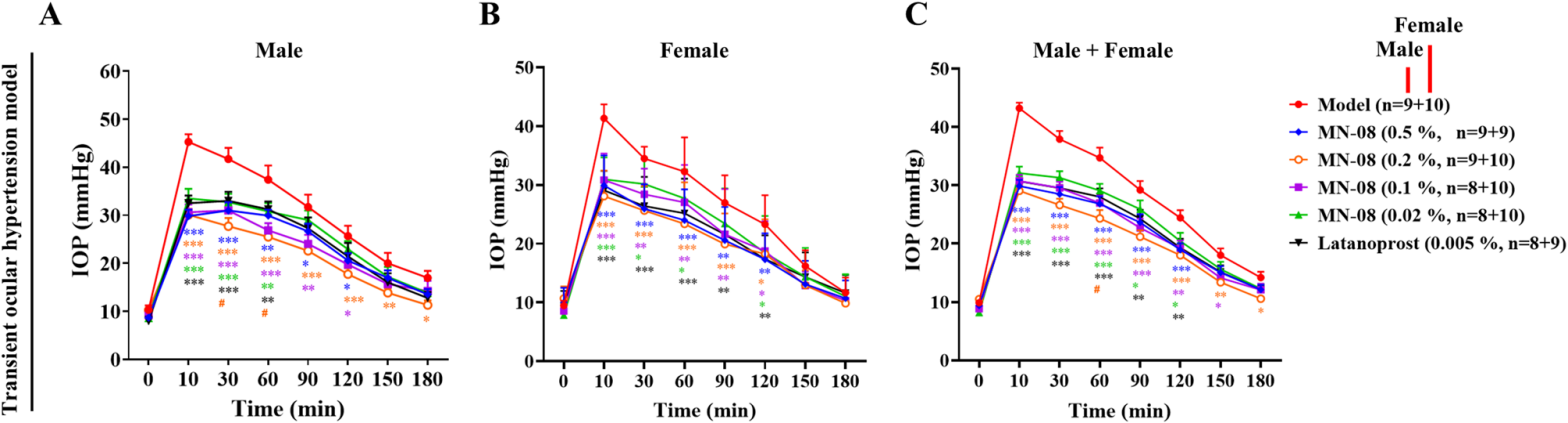
MN-08 lowers IOP in the transient ocular hypertension rabbit model. A transient ocular hypertension rabbit model was established by the injection of 5% NaCl solution (0.1 mL) into the vitreous body. Various concentrations of MN-08 or latanoprost at a single dose were then instilled just after the hypertonic saline solution injection. IOP was measured at baseline (time 0) and 10, 30, 60, 90, 120, 150, and 180 min post-drug administration. Data were presented as the means ± SEM. *P < 0.05 versus Model group; **P < 0.01 versus Model group; ***P < 0.001 versus Model group; ^*#*^*P* < 0.05 versus Latanoprost group.

The transient ocular hypertension model in rabbits was used to assess the effects of MN-08 on IOP. As shown in Fig. 3A–C, the injection of hypertonic saline solution into the vitreous body resulted in a transient IOP increase from baseline (9.87 ± 1.88 mmHg) to peak (43.21 ± 4.00 mmHg). After injection of hypertonic saline, IOP in both male and female rabbits increased rapidly, reached a maximum at 10 min, and subsequently began to decline, returning to baseline values within 180 min post-injection. Specifically, topically applied MN-08 at concentrations ranging from 0.02 to 0.5% elicited a significant decrease in IOP (in both males and females) throughout the experimental duration. The exposure to MN-08 led to rapid IOP reduction that reached its maximum within 10 to 90 min after a single dose. In particular, the effect of MN-08 at concentrations of 0.1, 0.2, and 0.5% was sustained up to 120 min.

### MN-08 lowers IOP in the carbomer-induced glaucoma rabbit model

**Fig. 4:**
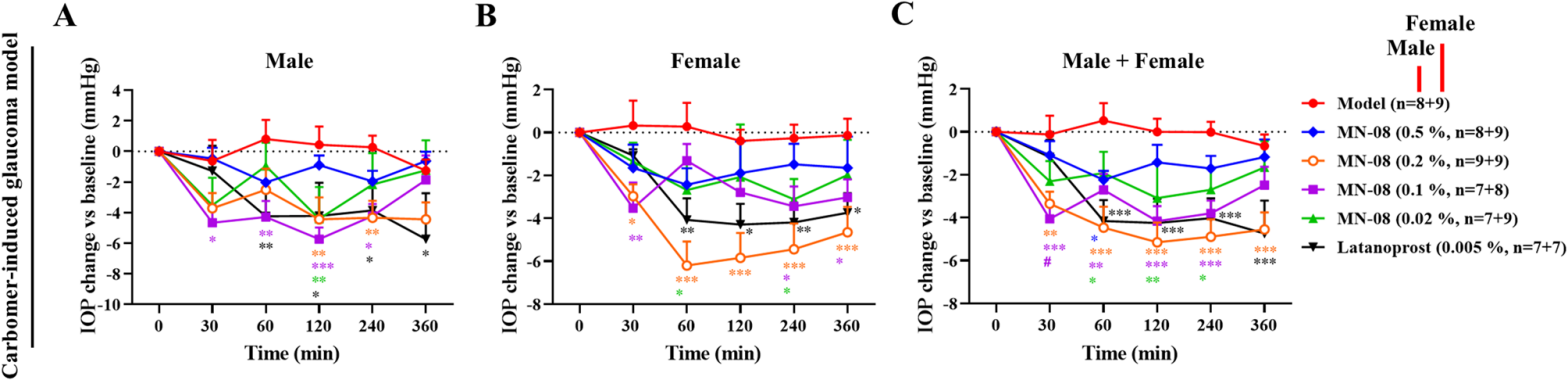
MN-08 lowers IOP in the carbomer-induced glaucoma rabbit model. Injection of 0.25% carbomer into the anterior eye chamber bilaterally to induce experimental glaucoma in rabbits. After MN-08 administration, IOP was measured at 0, 30, 60, 120, 240, and 360 min. The changes in IOP from baseline at different time points were calculated. Data were presented as the means ± SEM. *P < 0.05 versus Model group; **P < 0.01 versus Model group; ***P < 0.001 versus Model group; ^*#*^*P* < 0.05 versus Latanoprost group.

We further investigated whether MN-08 produced a similar IOP-lowering effect in the carbomer-induced glaucoma rabbit model. As previously described^38^, a water suspension of 0.25% commercially available carbomer was bilaterally injected into the anterior eye-chamber to induce experimental glaucoma in rabbits. As shown in Fig. 4A–C, MN-08 and latanoprost both reduced carbomer-induced IOP elevation to varying degrees at each time point after the initial IOP as the baseline value. Particularly in female rabbits, the 0.2% dose of MN-08 exhibited the most substantial reduction in IOP, with this IOP-lowering effect being sustained for 360 min. Taken together, these results indicated that MN-08 could significantly reduce IOP in the rabbit model of transient ocular hypertension and carbomer-induced glaucoma.

### MN-08 increases the cGMP levels in HTMCs

**Fig. 5:**
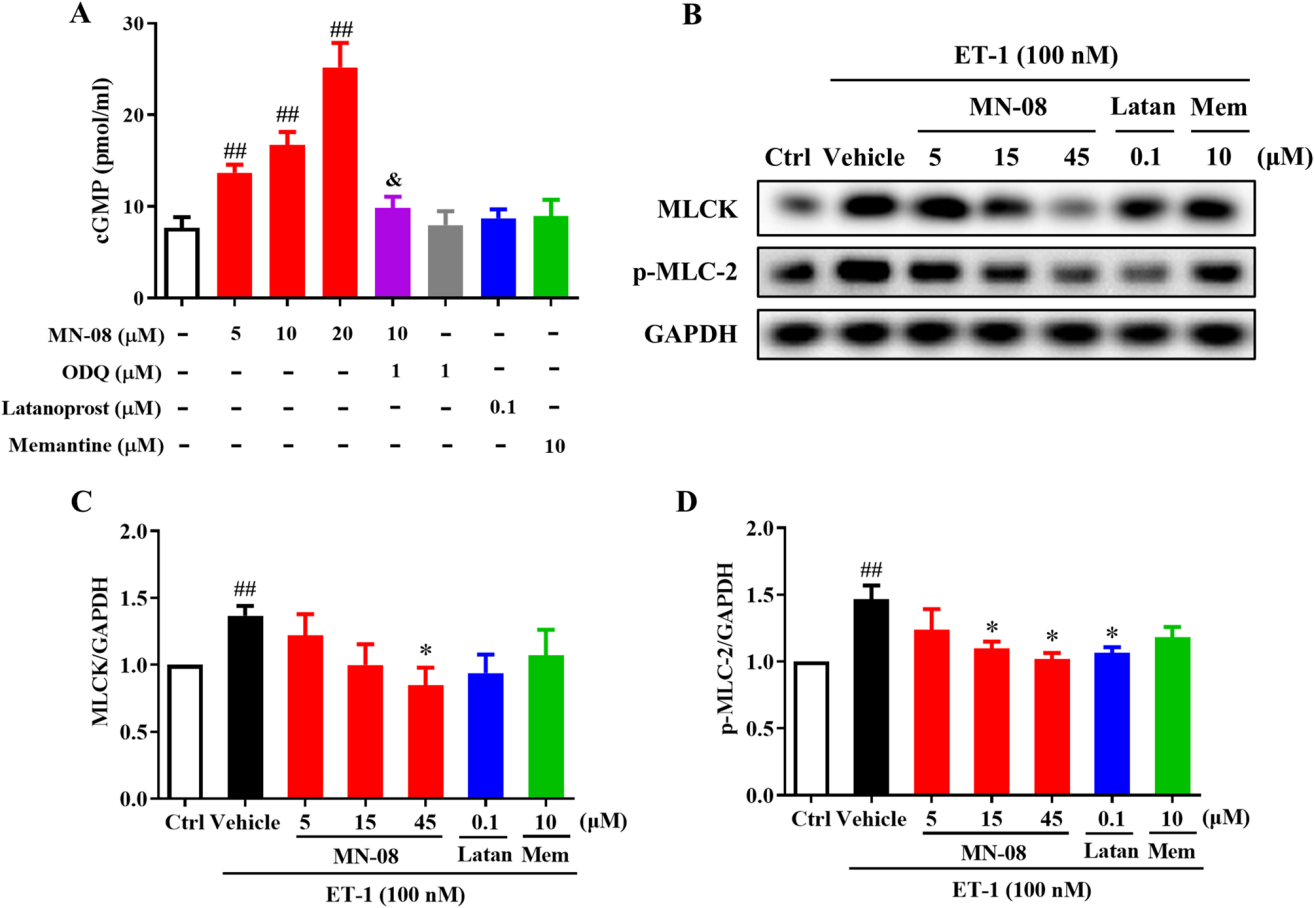
MN-08 increases cGMP levels and regulates the expression of MLCK and p-MLC-2 in HTMCs. (**A**) HTMCs were pretreated with ODQ (1 μM) for 30 min prior to incubation with varying concentrations of MN-08, latanoprost (Latan), and memantine (Mem). ELISA kit was used for detecting cGMP levels in cell lysates (n = 4). **(B**) HTMCs were preincubated with MN-08 at increasing concentrations for 60 min, and subsequently treated with either a vehicle or 100 nM ET-1 for 5 min. After the treatment period, the collected cell lysates were assessed by Western blot analysis with chemiluminescent detection. The relative expression of MLCK (**C**) and p-MLC-2 (D) was quantified in HTMCs (n = 3). Data were presented as the means ± SEM. ^*##*^*P* < 0.01 versus Ctrl group; ^*&*^*P* < 0.05 versus MN-08 (10 μM) group; *P < 0.05 versus Vehicle group.

The trabecular meshwork (TM) serves as a primary target for NO donors. It has been demonstrated that these donors facilitate the relaxation of TM by activating selective soluble guanylyl cyclase (sGC) and increasing cGMP levels, which subsequently enhances conventional outflow facilities. We determined that MN-08 exerts its relaxing effect on TM by releasing NO by measuring the cGMP levels in HTMCs. As shown in Fig. 5A, MN-08 (5, 10, 20 μM) significantly elevated cGMP levels in a concentration-dependent manner. In contrast, neither latanoprost (0.1 μM) nor memantine (10 μM) had any statistically significant effects on cGMP levels. To further confirm the involvement of the sGC pathway in the cGMP increase induced by MN-08, HTMCs were pretreated with the specific sGC inhibitor ODQ (1 μM) before MN-08 administration. As expected, ODQ eliminated the effect of MN-08 (10 μM) in elevating cGMP. These results indicated that MN-08 induces cGMP elevation by releasing NO and that this effect is mediated by sGC.

### MN-08 dephosphorylates MLC-2 and mediates MLCK expression in HTMCs

The trabecular meshwork also exhibits smooth-muscle-like properties and plays a crucial role in regulating AH outflow and IOP. Cellular contraction in this context is mediated by actin/myosin interactions, which necessitate the phosphorylation of myosin light chain (MLC). To further evaluate the effect of MN-08 on regulating HTMCs contractility and underlying signaling pathways in TM through releasing NO, Endothelin-1 (ET-1) was used to induce HTMC contractility, and the phosphorylation of MLC-2 (p-MLC-2) was determined by Western blotting. As shown in Fig. 5B, ET-1 (100 nM) markedly increased the protein expression of MLCK and p-MLC-2 in HTMCs. MN-08 (45 μM) pretreatment resulted in a substantial reduction in the protein levels of MLCK and p-MLC-2 (Fig. 5C–D), demonstrating a more pronounced effect than that observed with memantine and latanoprost. MN-08 provides an additional effect in decreasing HTMC ET-1-induced p-MLC-2 compared to memantine. These findings indicated that the NO-donating moiety of MN-08 mediates HTMC relaxation through activation of the cGMP signaling pathway and a subsequent reduction in MLC-2 phosphorylation.

### Tissue distribution and pharmacokinetic parameters of MN-08 in rabbit ocular tissues

**Fig. 6:**
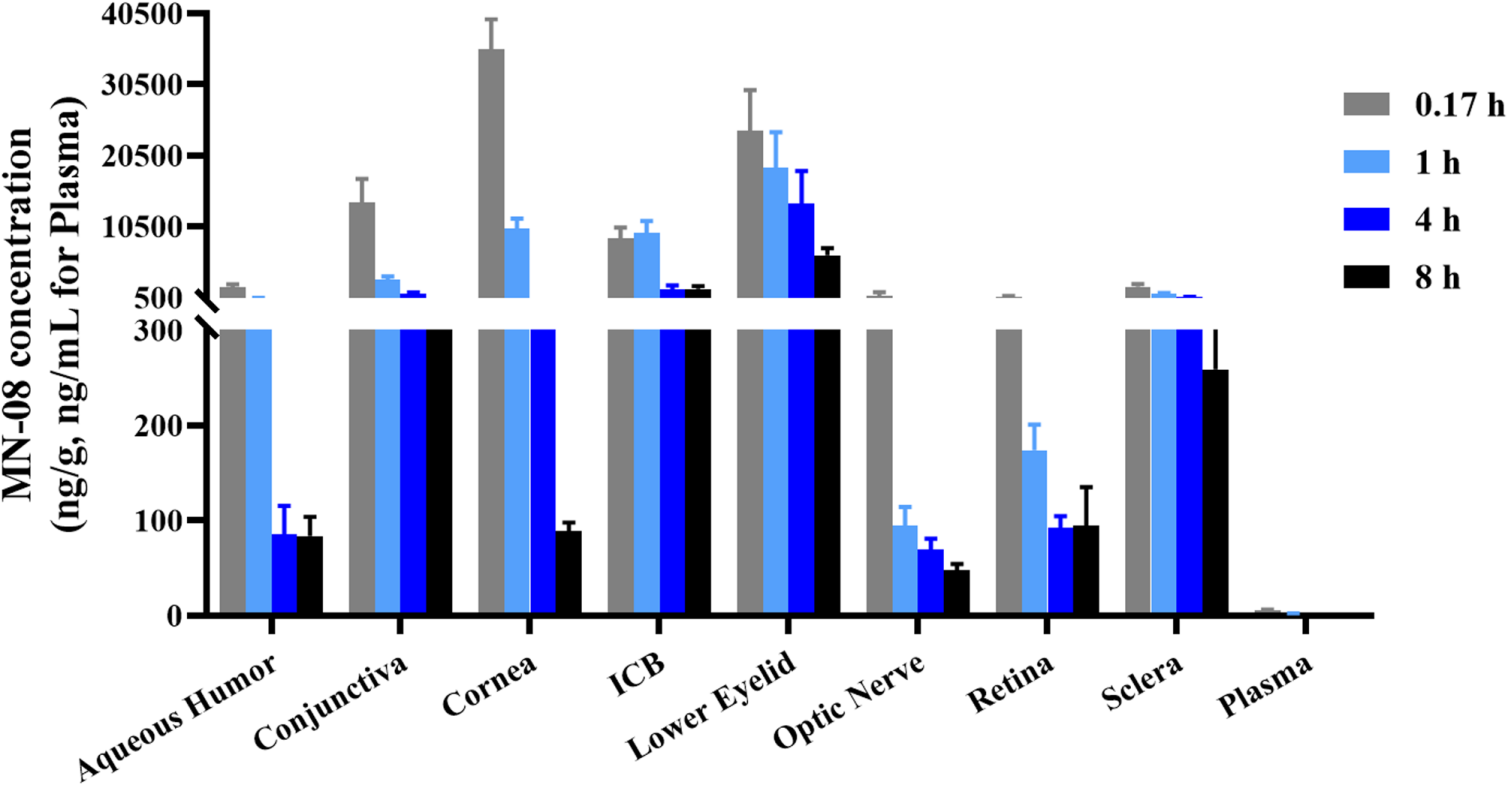
Tissue distribution of MN-08 in rabbit ocular tissues at different time points after topical instillation. Data were presented as the means ± SEM (n = 6).

The objective of this experiment was to study the distribution characteristics and pharmacokinetic parameters of MN-08 in rabbit ocular tissues after topical instillation. As shown in Fig. 6, MN-08 could disperse into various ocular tissues of rabbits through the cornea after topical instillation. The concentration of MN-08 in the cornea, aqueous humor, iris-ciliary body (ICB) and retina of rabbits are 32,516.46, 1482.00, 8934.34, and 774.63 ng/mL (equivalent to111.82, 5.01, 30.72 and 2.66 µM, respectively) following a single topical instillation of MN-08 at a dose of 0.25 mg/eye, respectively (Table 1). In addition, the concentration of MN-08 in plasma was very low after topical administration of MN-08 in the eye. Maximum concentration (C_max_) was observed in the cornea and aqueous humor at 0.17 h post-administration, suggesting that rapid absorption of MN-08 into the aqueous humor of the eye following topical instillation.

**Table 1:** Pharmacokinetic parameters of MN-08 in rabbit ocular tissues after topical instillation.

Tissue	T_max_ (h)	C_max_ (ng/g)	AUC_last_ (h ng/g)
Aqueous humor	0.17	1482.00	1988.74
Conjunctiva	0.17	11,943.92	17,905.76
Cornea	0.17	32,516.46	35,642.25
Iris-ciliary body (ICB)	0.17	8934.34	31,213.37
Lower eyelid	0.17	20,042.83	97,526.39
Optic nerve	0.17	437.92	782.77
Retina	0.17	774.63	2474.64
Sclera	0.17	1974.21	5411.39
Plasma	0.17	5.22 (ng/mL)	7.05 (h ng/mL)

T_max_, C_max_ and AUC_last_ values are presented as means. *ICB* iris-ciliary body, *C*_*max*_:maximum concentration, *T*_*max*_ time of maximal concentration, *AUC*_*last*_ area under the curve.

## Discussion

Glaucoma is the most common cause of blindness in adult, affecting approximately 80 million individuals globally. It is characterized by reduced aqueous humor flow and elevated IOP, and is closely related to degenerative optic neuropathy and RGCs loss^39^. Currently, the management of glaucoma primarily focuses on IOP control and neuroprotection^40^. While IOP reduction delays disease progression, it fails to address pre-existing and ongoing neurodegenerative changes in the optic nerve. A multifaceted therapeutic strategy is required to actively support, protect, and potentially restore RGCs and optic nerve integrity. Neuroprotective approaches are critical for overcoming the limitations of current treatment paradigms, as they target fundamental pathological mechanisms beyond IOP modulation^41^.

Memantine is a non-competitive NMDA antagonist with anti-glutamate excitotoxicity effects. Though typically indicated in moderate to severe AD, memantine has also been shown to reduce glutamate-induced excitotoxicity and protect against RGC loss in animal models of glaucoma^42–44^. However, memantine failed to significantly delay glaucoma progression in clinical trials^45^. In the present study, we found that MN-08, a novel compound exhibiting a dual mechanism of action through its NO-releasing and memantine moieties, effectively lowered IOP and prevented RGCs loss in established rat and rabbit models of glaucoma, suggesting that MN-08 has the potential to be a promising candidate for anti-glaucoma therapy.

Elevated intraocular pressure (IOP) is one of the major risk factors for developing primary open-angle glaucoma, accounting for 80% of glaucoma cases. Pathologically elevated IOP is a major cause of damaged vision and RGCs injury, recognized as a significant risk factor for the advancement of optic neuropathy^46^. Thus, prompt IOP-lowering therapy remains an important direction in preventing the development of glaucoma or slowing its progression. In this work, hypertonic saline and carbomer were used to induce a transient ocular hypertension and an experimental glaucoma rabbit model; these models are considered suitable for studying IOP-lowering effects in glaucoma. MN-08 showed significant IOP-lowering effects in both models described above.

IOP is primarily regulated by the trabecular meshwork (TM), a specialized structure in the ocular anterior segment that drains approximately 80–90% of aqueous humor (AH)^47^. Prolonged IOP elevation leads to RGC axonal damage and functional defects, resulting in progressive deterioration of the patient’s vision^48^. Nitric oxide (NO) directly regulates IOP by modulating the dynamic equilibrium between the rates of production and drainage of AH, as well as the impairment of the NO-guanylate cyclase (GC) pathway^49^. The trabecular meshwork (TM) and Schlemm’s canal (SC) are predominant sites of NO action, both of which control the resistance to outflow of AH and thus IOP^50^. Our previous research indicated that MN-08 can bind to NMDA receptors, releasing NO, which dilates cerebral blood vessels and increases cerebral blood flow, subsequently ameliorating brain injury and cerebral vasospasm^32^. In the present study, the role of MN-08 in reducing IOP may be attributed to its ability to release NO in ocular tissues, which facilitates TM relaxation and ultimately enhances conventional outflow. As expected, we found that MN-08 was able to mediate HTMC relaxation by activating the NO/cGMP signaling pathway and subsequently reducing MLC-2 phosphorylation. These data indicate that MN-08 regulates the contraction and relaxation of the TM cells, increasing AH outflow and lowering IOP.

Glaucoma is a multifactorial neurodegenerative disease with a complex pathogenesis^51^. Although high IOP is recognized as a major risk factor for glaucoma, traditional drugs that solely reduce IOP have proven to be unable to prevent or reverse progressive RGCs loss and visual field deficits in some glaucoma patients. Increasing evidence shows that the focus of glaucoma research may shift towards neuroprotection, as there are numerous similarities among glaucoma and other central nervous system degenerative diseases^16,52^. Excitotoxicity induced by elevated glutamate levels, amyloid beta peptide aggregation, and oxidative stress damage play an important role in the development of glaucomatous neurodegeneration, as they have in Alzheimer’s disease^53^. Glutamate-mediated excitotoxicity, primarily through NMDA receptors, is closely related to RGCs death in glaucoma. Intracellular calcium serves as a significant mediator of RGC death^54^. Elevated levels of released glutamate excessively stimulate glutamate receptors, resulting in apoptosis through a significant influx of Ca^2+^ into cells, predominantly via NMDA receptors. It is therefore believed that pharmacological inhibition of NMDA receptors is a critical strategy for neuroprotection in glaucoma. Previous research has reported that MN-08 can bind to and inhibit the over-activation of neuronal NMDA receptors, effectively blocking Ca^2+^ influx and thereby reducing glutamate-mediated excitotoxic neuronal damage^32,33^. Moreover, MN‐08 also exhibits significant therapeutic effects in several neurodegenerative conditions due to its powerful neuroprotective effect, including Alzheimer’s disease (AD), vascular dementia (VD), and subarachnoid hemorrhage (SAH)^32,33^. In this work, we found that MN-08 treatment prevented RGCs loss and reversed the altered expression of apoptosis-related proteins in the retinas of acute glaucoma rats. We speculate that the benefits of MN-08 for RGCs loss in glaucoma may be attributed to its antagonization of NMDA receptors. These data indicate that MN-08 may be an effective neuroprotective agent for glaucoma. In addition, MN-08 exhibited favorable distribution characteristics and pharmacokinetic parameters in normal rabbit ocular tissues after topical instillation and was rapidly penetrated and absorbed into the aqueous humor and other ocular tissues.

In conclusion, our results demonstrated that an innovative compound MN-08 has therapeutic efficacy in multiple experimental glaucoma models. The advantages of the dual-functional MN-08 in neuroprotection and IOP reduction are likely to provide significant benefits in the treatment of patients with glaucoma. However, limitations of this study remain. It is necessary to further evaluate the therapeutic effects of MN-08 in axonal injury and long-term glaucoma models.

## Electronic supplementary material

Below is the link to the electronic supplementary material.

Supplementary Material 1

Supplementary Material 2

## Data Availability

Data is provided within the manuscript or supplementary information files.
