# Supplementary figures and images for: Neuroprotective and intraocular pressure lowering effects of dual-functional memantine nitrate MN-08 on the experimental models of glaucoma
Source: Sci Rep. 2025 Jul 3;15:23822. doi: 10.1038/s41598-025-06832-x (PMC12229680; doi:10.1038/s41598-025-06832-x)

Figure 2A

**
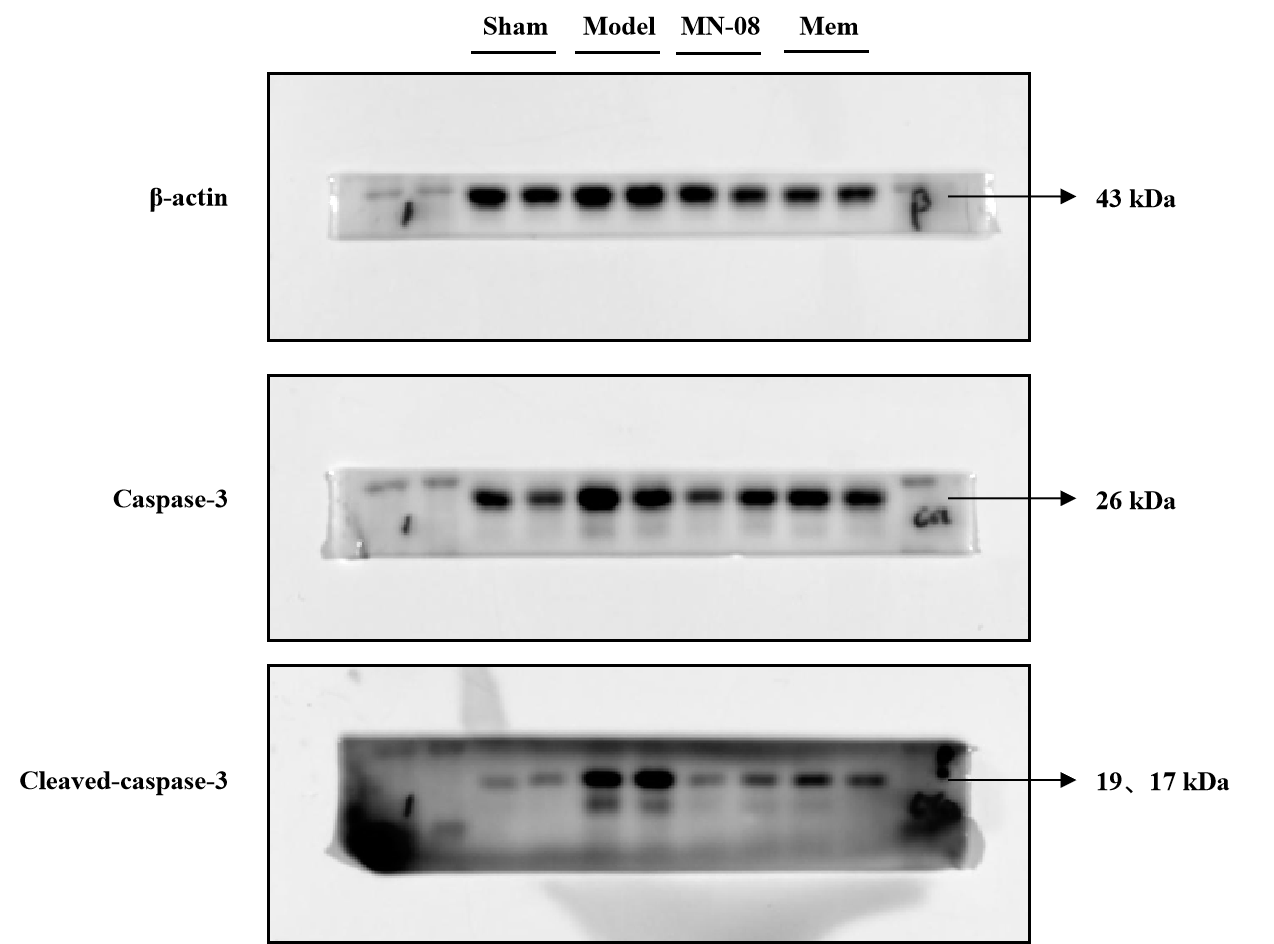
**

Figure 2B

**
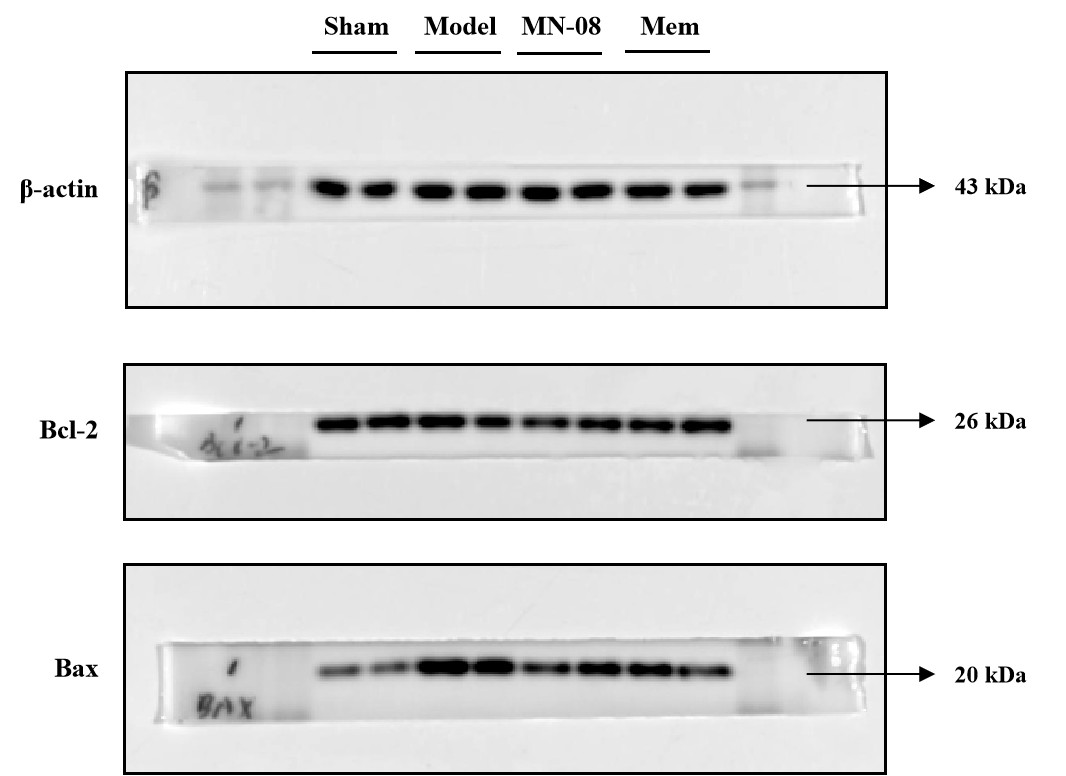
**

Figure 5B

**
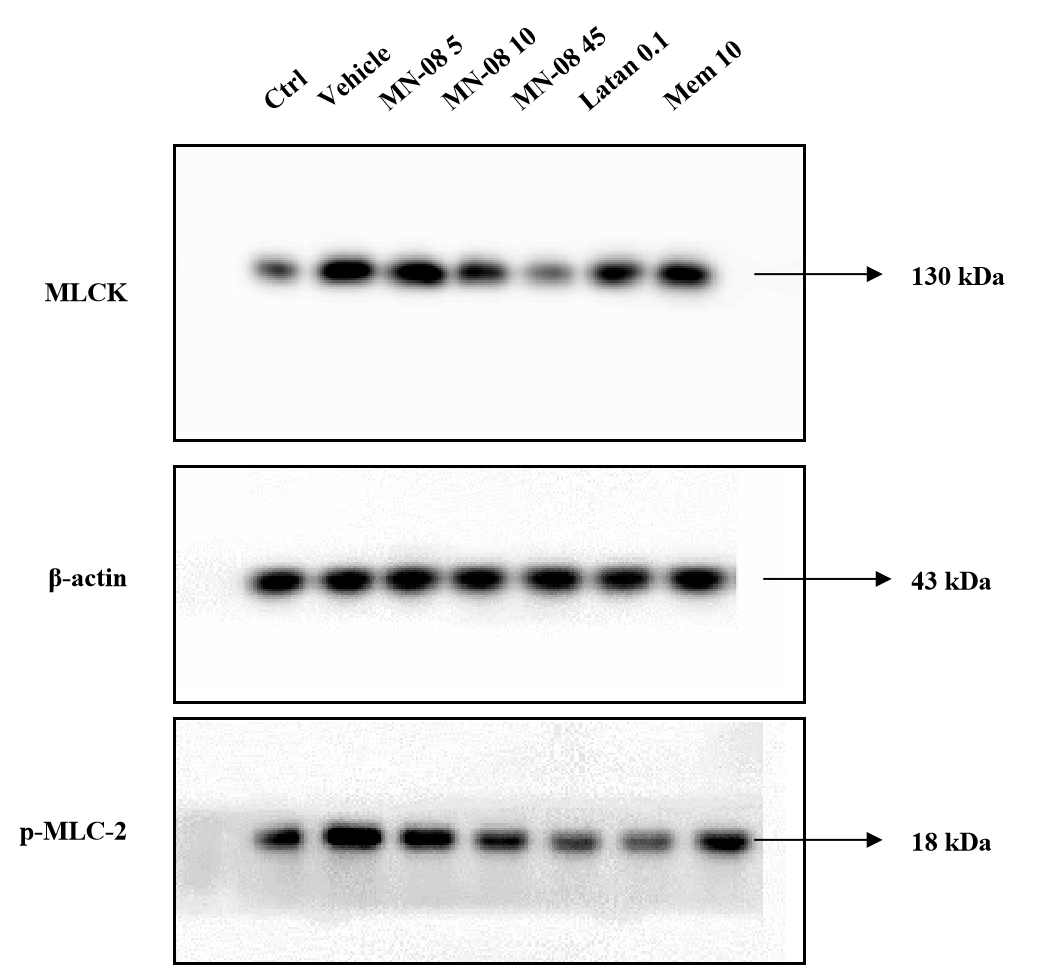
**

Supplement: Supplementary file 1 — Supplementary Material 1 [file 41598_2025_6832_MOESM1_ESM.docx]
