# Supplementary material for: Neuroprotective and intraocular pressure lowering effects of dual-functional memantine nitrate MN-08 on the experimental models of glaucoma
Source: Sci Rep. 2025 Jul 3;15:23822. doi: 10.1038/s41598-025-06832-x (PMC12229680; doi:10.1038/s41598-025-06832-x)
Supplement: Supplementary file 2 — Supplementary Material 2 [file 41598_2025_6832_MOESM2_ESM.docx]

**Neuroprotective and intraocular pressure lowering effects of dual-functional memantine nitrate MN-08 on the experimental models of glaucoma**

Huihui Hu ^a, b, c, d, †^, Liangmiao Wu ^a, b, c, d, †^, Xiaodie Hu ^b, c, d^, Minghua Wang^e^, Yewei Sun ^b, c, d^, Gaoxiao Zhang ^b, c, d^, Yuqiang Wang ^b, c, d^, Peng Yi ^b, c, d, *^, Zaijun Zhang ^b, c, d, *^

**Supplemental Figure S1**

**
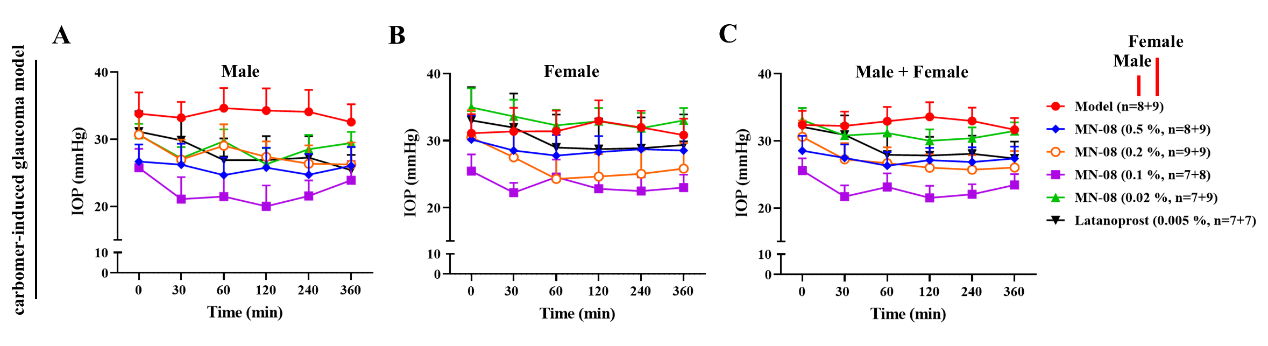
**

**Fig. S1.** MN-08 lowers IOP in the carbomer-induced glaucoma rabbit model. Injection of 0.25% carbomer into the anterior eye-chamber bilaterally to induce experimental glaucoma in rabbits. After MN-08 administration, IOP was measured at 0, 30, 60, 120, 240, and 360 min. Data were presented as the means ± SEM.
